# A Population-Based Study on Liver Metastases in Women With Newly Diagnosed Ovarian Cancer

**DOI:** 10.3389/fonc.2020.571671

**Published:** 2020-09-25

**Authors:** Haiyun Zhao, Fei Xu, Jiajia Li, Mengdong Ni, Xiaohua Wu

**Affiliations:** ^1^Department of Gynecologic Oncology, Fudan University Shanghai Cancer Center, Shanghai, China; ^2^Department of Oncology, Shanghai Medical College, Fudan University, Shanghai, China

**Keywords:** liver metastases, ovarian cancer, incidence, prognosis, SEER

## Abstract

**Aim:** The purpose of this study was to analyze the incidence, clinical characteristics, prognostic factors and survival of ovarian cancer patients with liver metastases upon initial diagnosis.

**Methods:** Patients with ovarian cancer liver metastases upon initial diagnosis between 2010 and 2016 were identified from the Surveillance, Epidemiology, and End Results (SEER) database. Univariate and multivariate logistic regression was performed to identify the predictors of the presence of liver metastases in newly diagnosed ovarian cancer patients. Overall survival (OS) was assessed using the Kaplan-Meier method and log-rank test. Univariate and multivariate Cox regression was conducted to determine the independent prognostic factors for OS.

**Results:** A total of 1,744 ovarian cancer patients with liver metastases was identified from the SEER database, accounting for 6.7% of the entire ovarian cancer patients. As to the unique distant organ provided by SEER, liver was the most common metastatic site of ovarian cancer (4.65%). Age, race, laterality, histology, pathological grade, extrahepatic sites, stage of tumor were the predictors of the presence with liver metastases revealed by multivariable logistic regression model. Median OS for the patients with liver metastases at initial diagnosis of ovarian cancer was 16.0 months. Multivariate Cox regression model confirmed race, histology, extrahepatic metastatic sites, surgery and marital status were independent prognostic factors for OS.

**Conclusion:** The study provided population-based estimates of the incidence and prognosis of newly diagnosed ovary cancer patients with liver metastases, which could be potentially used for the risk assessment and individualized treatment.

## Introduction

According to the latest cancer statistics in 2019, there were about 22,530 patients newly diagnosed with ovarian cancer and 13,980 patients died of ovarian cancer. Ovarian cancer accounts for 2.5% of all cancers in women and ranks the fifth cause of cancer death among women in the United States (1). Owing to the scarcity of early, specific symptoms and effective screening strategies, patients with ovarian cancer were often diagnosed with synchronous distant metastases at an advanced stage, accounting for about 70% of the whole ovarian cancer population (2). The survival of ovarian cancer patients was stage-dependent. The 5 year survival was 92, 75, 29%, respectively, for the localized cases, regional cases and distant cases (1). Studies of the patterns of distant metastases in stage IV ovarian cancer showed liver was the most common distant metastatic organ of ovarian cancer with the proportion of 37–57%, followed by distant lymph nodes, lung, bone and brain (3–5). Autopsy studies of cases died of primary ovarian cancer showed that the incidence of liver parenchyma was about 48.2–73% (5, 6). The median overall survival (OS) was 30 months for patients with a single liver metastasis (4).

At present, data of prevalence and prognostic factors among ovarian cancer patients with liver metastases is limited. The therapy for ovarian cancer patients with liver metastases is controversial. Here, we explored the Surveillance, Epidemiology, and End Results (SEER) database to analyze the characteristics, incidence, risk factors and prognostic factors of ovarian cancer patients with liver metastases upon initial diagnosis.

## Materials and Methods

### Study Population

All the primary data were acquired from the SEER database, which collected the information of patient demographics, tumor characteristics, treatment and prognosis accounting for ~30% of the whole population in the United States. The datasets of this current study are available from SEER^*^Stat Version 8.3.6 (http: https://seer.cancer.gov/seerstat/). Patients diagnosed with primary and microscopically confirmed ovarian cancer as the only primary malignancy between 1 January 2010 and 31 December 2016 were included in the study. We excluded those patients younger than 18 years old, diagnosed with carcinoma *in situ*, benign or borderline tumors, with unknown information of liver metastases, diagnosed by autopsy or death certificate. Finally, there were 26,197 patients eligible for incidence analysis. Among these patients, 1,744 patients had liver metastases upon initially diagnosis of ovarian cancer. Next, we removed patients with unknown follow-up, as well as patients who presented with 0 day of survival record, leaving 25,819 patients and 1,718 patients with liver metastases for the survival analysis.

### Statistical Analysis

The incidence of different distant metastatic organs including liver, bone, brain, and lung among the total patients with ovarian cancer was calculated in this study. Incidence was defined the number of ovarian cancer patients with corresponding metastases divided by the whole number of patients with ovarian cancer.

We compared the patient characteristics between patients with liver metastases and those without liver metastases by the Pearson chi-square test or Fisher's exact test appropriately. Study variables in the descriptive statistics were included as follows: age at diagnosis, year of diagnosis, race, laterality, histology, primary tumor stage, regional lymph node stage, pathological grade, surgery, numbers of extrahepatic metastatic sites (bone, brain, and lung), marital status and insurance status. In the SEER database, tumor grades were classified into I (well-differentiated), II (moderately differentiated), III (poorly differentiated), and IV (undifferentiated or anaplastic).

Univariable and multivariable logistic regression was conducted to determine the risk factors for the liver metastases at initial diagnosis of ovarian cancer. Variables including age at diagnosis, race, laterality, histology, pathological grade, the extent of extrahepatic metastatic sites, primary tumor stage, lymph node stage, marital status and insurance status were analyzed in this model. Odds ratios (OR) and 95% confidence intervals (CI) were reported from the logistic regression.

Overall survival (OS) was determined as the date of diagnosis of ovarian cancer to the date of death due to any cause or the date of last follow-up. We utilized the univariable and multivariable Cox proportional hazard models to assess the association of study variables with OS by reporting the hazard ratios (HR) and 95% Cis. In our study, the marital status was enrolled in the analysis of prognostic factors for the following reasons. First, epidemiological studies have reported a reduced risk of ovarian cancer among women who have had children. And infertility has been associated with an increasing risk of ovarian cancer (7, 8). Second, married patients with cancer may have more support from family members, social services and insurance than unmarried patients. Unmarried patients are at significant higher risk of undertreatment and death from cancer (9). Kaplan-Meier method was performed to obtain the survival estimates and the log-rank test was conducted to analyze the difference between subgroups.

All the statistical analyses were performed by the SPSS statistical software version 22 (IBM Corporation, USA). All *P*-values calculated were two-sided, and a *p* < 0.05 was considered statistically significant.

## Results

### Incidence

**Figure 1:**
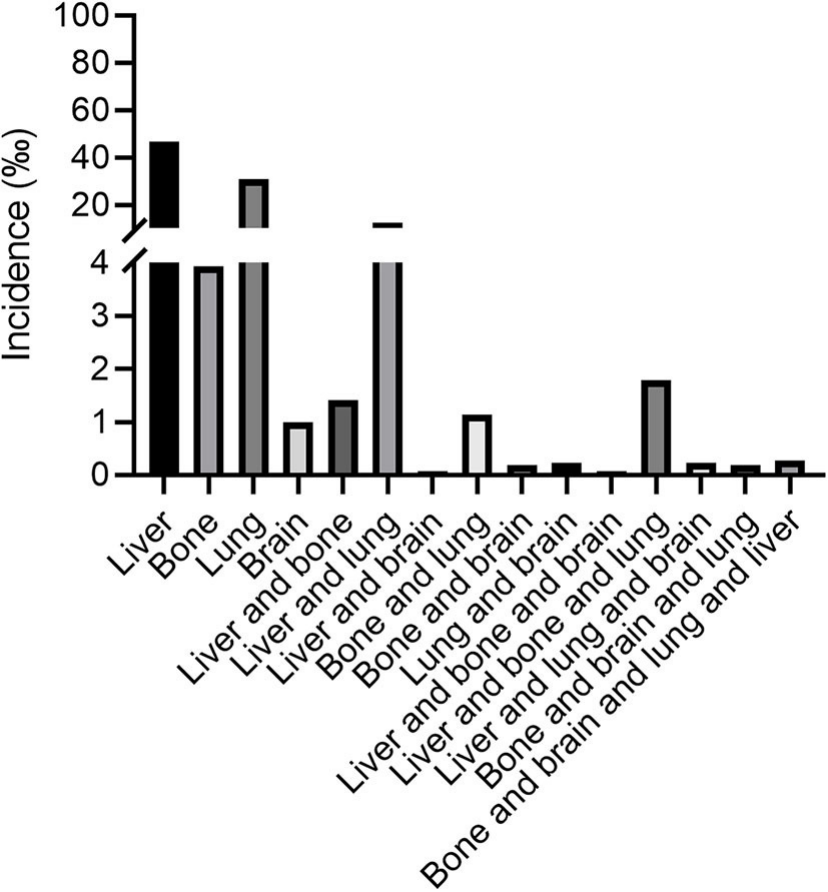
The incidence of ovarian cancer patients with liver, lung, bone, and brain metastases at the time of initial diagnosis.

Total of 26,197 patients were diagnosed with ovarian cancer from 2010, January to 2016, December reported by the SEER database. Among the total 26,197 patients, there were 13,366 patients with serous ovarian cancer (51.0%), 2,321 patients with endometrioid carcinoma (8.9%), 1,765 patients with clear cell ovarian cancer (6.7%), 1,587 patients with mucinous carcinoma (6.1%) and 7,158 patients with other types such as granular cell cancer and Brenner tumor (27.3%). In view of the small size, we categorized the non-serous ovarian cancer into one group. Figure 1 illustrated the incidence of patients with distant metastases at initial diagnosis of ovarian cancer according to the metastatic sites including liver, bone, lung and brain among the entire cohort. 4.65, 0.39, 4.00, 0.10% ovarian cancer patients had liver metastases, bone metastases, lung metastases, brain metastases only, respectively, at initial diagnosis. As to patients with two distant metastatic sites, patients with liver and lung metastases had the highest proportion, accounting for 1.20% among the entire population.

### Patient Characteristics

One thousand seven hundred and forty four patients had liver metastases at initial diagnosis of ovarian cancer, accounting for 6.7% among the total population. Table 1 showed the demographic and clinical characteristics for ovarian cancer patients with and without liver metastases. Compared to patients without liver metastases at initial diagnosis, patients with liver metastases were older (*P* < 0.001), were more likely to be Black (*P* < 0.001), had bilateral tumors (*P* < 0.001), presented with more advanced T stage (*P* < 0.001), presented with more advanced N stage (*P* < 0.001), had a higher pathological grade (*P* < 0.001), had no surgery of primary tumor (*P* < 0.001), had more extrahepatic metastatic sites to lung, bone and brain (*P* < 0.001), were more likely to be unmarried (*P* < 0.001).

**Table 1:** Clinicopathological characteristics of ovarian cancer patients with and without liver metastases

**Patient characteristics**	**No. of ovarian cancer patients**				**Total**	***P*-value**
	**With liver metastases**		**Without liver metastases**			
	***N***	**%**	***N***	**%**	***N***	
All Patients	1,744	6.7	24,453	93.3	26,197	
Age at diagnosis						<0.001*
18–40	86	3.5	2,384	96.5	2,470	
41–60	594	5.5	10,219	94.5	10,813	
>60	1,064	8.2	11,850	91.8	12,914	
Year of diagnosis						0.592
2010	238	6.6	3,379	93.4	3,617	
2011	249	6.8	3,422	93.2	3,671	
2012	249	6.7	3,482	93.3	3,731	
2013	235	6.4	421	93.6	3,656	
2014	249	6.5	3,564	93.5	3,813	
2015	249	6.3	3,730	93.7	3,979	
2016	275	7.4	3,455	92.6	3,730	
Race						<0.001*
White	1,390	6.6	19,740	93.4	21,130	
Black	209	9.1	2,084	90.9	2,293	
Asian/Pacific islander	126	5.2	2,311	94.8	2,437	
American India/Alaskan	14	8.0	162	92.0	176	
Unknown	5	3.1	156	96.9	161	
Laterality						<0.001*
Left	270	3.8	6,770	96.2	7,040	
Right	317	4.3	6,999	95.7	7,316	
Bilateral	652	7.3	8,225	92.7	8,877	
Unknown	505	17	2,459	83	2,694	
Histology						0.201
Serous	864	6.5	12,502	93.5	13,366	
Non-Serous	880	6.9	11,951	93.1	12,831	
T stage						<0.001*
T1	54	0.8	6,341	99.2	6,395	
T2	106	3.6	2,876	96.4	2,982	
T3	1052	8.9	10,779	91.1	11,831	
Unknown	532	10.7	4,457	89.3	4,989	
N stage						<0.001*
N0	674	4.2	15,259	95.8	15,933	
N1	508	10.4	4,376	89.6	4,884	
Unknown	562	10.4	4,818	89.6	5,380	
Pathological grade						<0.001*
I	20	1	1,960	99	1,980	
II	75	2.6	2,761	97.4	2,836	
III	488	6.4	7,160	93.6	7,648	
IV	322	5.4	5,679	94.6	6,001	
Unknown	839	10.9	6,893	89.1	7,732	
Surgery						<0.001*
No	743	21.3	2,745	78.7	3,488	
Yes	998	4.4	21,696	95.6	22,694	
Unknown	3	20	12	80	15	
Extrahepatic metastatic sites						<0.001*
to lung, bone and brain, No.						
0	1,213	4.9	23,356	95.1	24,569	
1	364	28.1	933	71.9	1,297	
2	55	57.3	41	42.7	96	
All 3	7	58.3	5	41.7	12	
Unknown	105	47.1	118	52.9	223	
Marital status						<0.001*
Unmarried^a^	888	7.5	10,973	92.5	11,861	
Married	779	5.9	12,374	94.1	13,153	
Unknown	77	6.5	1,106	93.5	1,183	
Insurance status						0.952
Insured	1,654	6.7	23,204	93.3	24,858	
Uninsured	62	6.6	879	93.4	941	
Unknown	28	7	370	93	398	

a Including divorced, separated, single (never married), and widowed. *denotes a statistically significant P-value.

The results of univariable and multivariable logistic regression performed among the whole population was displayed in Table 2. On the univariable logistic regression model, age 41–60 years (vs. age 18–40 years, *P* < 0.001), age greater than 60 years (vs. age 18–40 years, *P* < 0.001), Black race (vs. White race, *P* < 0.001), bilateral (vs. left, *P* < 0.001), pathological grade II (vs. grade I, *P* < 0.001), grade III (vs. grade I, *P* < 0.001), grade IV (vs. grade I, *P* < 0.001), 1 extrahepatic site (vs. 0 extrahepatic site, *P* < 0.001), 2 extrahepatic sites (vs. 0 extrahepatic site, *P* < 0.001), 3 extrahepatic sites (vs. 0 extrahepatic site, *P* < 0.001), T2 stage (vs. T1 stage, *P* < 0.001), T3 stage (vs. T1 stage, *P* < 0.001), N1 stage (vs. N0 stage, *P* < 0.001) were significantly associated with greater odds of liver metastases at initial diagnosis of ovarian cancer. Asian/Pacific islander (vs. White race, *P* = 0.007), married status (vs. unmarried, *P* < 0.001) were associated with lower odds of liver metastases at diagnosis. Histology and insurance state were not associated with the risk of having liver metastases at initial diagnosis of ovarian cancer. On the multivariable logistic regression, age >60 years (vs. age 18–40 years, *P* = 0.032), Black race (vs. White race, *P* = 0.037); bilateral (vs. left, *P* < 0.001), non-serous type (vs. serous type, *P* < 0.001), grade III (vs. grade I, *P* < 0.001), grade IV (vs. grade I, *P* < 0.001), 1 extrahepatic site (vs. 0 extrahepatic site, *P* < 0.001), 2 extrahepatic sites (vs. 0 extrahepatic site, *P* < 0.001), 3 extrahepatic sites (vs. 0 extrahepatic site, *P* < 0.001), T2 stage (vs. T1 stage, *P* < 0.001), T3 stage (vs. T1 stage, *P* < 0.001), N1 stage (vs. N0 stage, *P* < 0.001) were significantly associated with greater odds of liver metastases at initial diagnosis of ovarian cancer. Married status (vs. unmarried status, *P* = 0.016) was associated with lower risk of liver metastases at initial diagnosis of ovarian cancer.

**Table 2:** Univariable and Multivariable logistic regression for the presence of liver metastases at diagnosis of ovarian cancer.

**Variables**	**Univariable**		**Multivariable**	
	**OR (95% CI)**	***P*-value**	**OR (95% CI)**	***P*-value**
**Age at diagnosis, y**				
18–40	1 (Reference)		1 (Reference)	
41–60	1.611 (1.280–2.029)	<0.001*	1.208 (0.943–1.548)	0.134
>60	2.489 (1.989–3.114)	<0.001*	1.307 (1.023–1.669)	0.032*
**Race**				
White	1 (Reference)		1 (Reference)	
Black	1.424 (1.223–1.658)	<0.001*	1.193 (1.011–1.409)	0.037*
Asian/Pacific islander	0.774 (0.642–0.934)	0.007*	0.892 (0.729–1.091)	0.265
American India/Alaskan	1.227 (0.709–2.124)	0.464	1.277 (0.709–2.300)	0.415
Unknown	0.455 (0.187–1.111)	0.084	0.637 (0.252–1.612)	0.341
**Laterality**				
Left	1 (Reference)		1 (Reference)	
Right	1.136 (0.962–1.340)	0.132	1.095 (0.921–1.302)	0.303
Bilateral	1.988 (1.719–2.299)	<0.001*	1.374 (1.173–1.610)	<0.001*
Unknown	5.149 (4.411–6.012)	<0.001*	1.818 (1.528–2.164)	<0.001*
**Histology**				
Serous	1 (Reference)		1 (Reference)	
Non-serous	1.065 (0.967–1.174)	0.201	1.449 (1.291–1.627)	<0.001*
**Pathological grade**				
I	1 (Reference)		1 (Reference)	
II	2.662 (1.620–4.374)	<0.001*	1.816 (1.094–3.014)	0.021
III	6.679 (4.259–10.475)	<0.001*	2.876 (1.812–4.563)	<0.001*
IV	5.557 (3.527–8.754)	<0.001*	2.435 (1.525–3.886)	<0.001*
Unknown	11.928 (7.634–18.638)	<0.001*	3.861 (2.441–6.107)	<0.001*
**Extrahepatic metastatic sites, No**.				
0	1 (Reference)		1 (Reference)	
1	7.512 (6.569–8.591)	<0.001*	4.590 (3.986–5.285)	<0.001*
2	25.830 (17.168–38.862)	<0.001*	16.249 (10.520–25.098)	<0.001*
All 3	26.957 (8.543–85.057)	<0.001*	14.152 (4.366–45.871)	<0.001*
Unknown	17.133 (13.090–22.426)	<0.001*	9.349 (7.047–12.403)	<0.001*
**T stage**				
T1	1 (Reference)		1 (Reference)	
T2	4.328 (3.109–6.024)	<0.001*	2.978 (2.118–4.188)	<0.001*
T3	11.460 (8.703–15.091)	<0.001*	6.642 (4.955–8.905)	<0.001*
Unknown	14.016 (10.566–18.592)	<0.001*	5.555 (4.020–7.675)	<0.001*
**N stage**				
N0	1 (Reference)		1 (Reference)	
N1	2.628 (2.331–2.963)	<0.001*	1.646 (1.448–1.872)	<0.001*
Unknown	2.641 (2.350–2.967)	<0.001*	1.421 (1.204–1.677)	<0.001*
**Marital status**				
Unmarried^a^	1 (Reference)		1 (Reference)	
Married	0.778 (0.704–0.859)	<0.001*	0.876 (0.786–0.976)	0.016*
Unknown	0.860 (0.676–1.095)	0.221	0.916 (0.705–1.190)	0.512
**Insurance status**				
Insured	1 (Reference)		1 (Reference)	
Uninsured	0.990 (0.761–1.286)	0.937	1.002 (0.754–1.332)	0.987
Unknown	1.062 (0.721–1.564)	0.762	1.011 (0.661–1.547)	0.959

a Including divorced, separated, single (never married), and widowed. *denotes a statistically significant P-value.

### Survival Analysis

**Figure 2:**
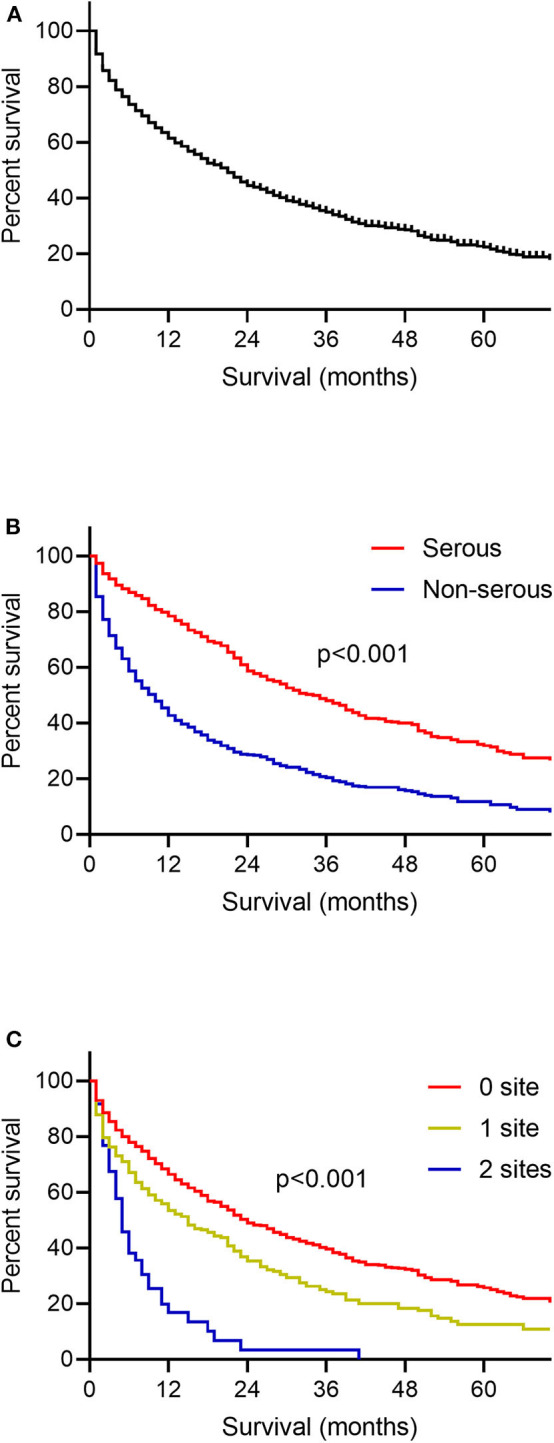
Kaplan-Meier curves for overall survival among patients with ovarian cancer liver metastases. **(A)** The whole population included in the survival analysis. **(B)** According to the histology type of ovarian cancer. **(C)** According to the number of extrahepatic metastatic sites to lung, bone and brain.

One thousand seven hundred and eighteen ovarian cancer patients with liver metastases were enrolled in the survival analysis. There were 854 patients with serous ovarian cancer (49.7%), 44 patients with endometrioid carcinoma (2.6%), 48 patients with clear cell ovarian cancer (2.8%), 46 patients with mucinous carcinoma (2.7%) and 726 patients with other types such as granular cell cancer and Brenner tumor (42.3%). The median OS among patients with liver metastases was 16.0 months, and the interquartile range (IQR) was 3.0–50.0 months (Figure 2A). OS curves stratified by ovarian cancer type of pathology was illustrated (Figure 2B). Patients with serous ovarian cancer had better survival than those with non-serous ovarian cancer (median OS: 30.0 months vs. 6.0 months, log-rank test, *P* < 0.001). The impact of the extent of extrahepatic metastatic disease on the survival of patients with liver metastases was estimated (Figure 2C, log-rank test, *P* < 0.001). Patients with more numbers of extrahepatic metastatic sites had worse prognosis.

Among the whole population, there were 1,385 patients with extrahepatic metastatic disease. The presence of liver metastases or not on the median survival of these patients stratified by the extent of extrahepatic metastatic sites was concluded in Table 3. Broadly speaking, patients with no baseline of liver metastases had better survival than patients with liver metastases. Specifically, significant difference was shown in patients with bone and liver metastases (median OS, 6.0 months; IQR, 1.0–10.0 months) vs. those with bone metastases only (median OS, 10.0 months; IQR, 3.0–44.0 months) (log-rank test, *P* = 0.003), patients with lung and liver metastases (median OS, 13.0 months; IQR, 2.0–33.0 months) vs. those with lung metastases only (median OS, 21.0 months; IQR, 6.0–41.0 months) (log-rank test, *P* = 0.001), patients with brain and liver metastases (median OS, 1.0 months; IQR, 1.0–1.0 months) vs. those with brain metastases only (median OS, 7.0 months; IQR, 2.0-NR) (log-rank test, *P* = 0.016).

**Table 3:** Median OS of ovarian cancer patients stratified by extrahepatic metastatic disease.

**Distant metastatic sites**	**Median OS (IQR), months**		***P*-value**
	**Liver and extrahepatic metastatic disease**	**Extrahepatic metastatic disease only**	
Bone	6.0 (1.0–10.0)	10.0 (3.0–44.0)	0.003*
Lung	13.0 (2.0–33.0)	21.0 (6.0–41.0)	0.001*
Brain	1.0 (1.0–1.0)	7.0 (2.0–NR)	0.016*
2 of 3	5.0 (2.0–9.0)	5.0 (2.0–24.0)	0.031*
All 3	2.0 (0.0–17.0)	7.0 (4.0–7.0)	0.958

IQR, interquartile range; NR, not reached. *denotes a statistically significant P-value.

Univariate and multivariate Cox proportional hazards models were performed to evaluate the prognostic factors of ovarian cancer patients with liver metastases (Table 4). In the univariate Cox models, Black race (vs. White race, HR,1.500; 95% CI, 1.261–1.784; *P* < 0.001); American India/Alaskan (vs. White race, HR, 1.776; 95% CI, 1.027–3.071; *P* = 0.04), non-serous type(vs. serous type, HR, 2.493; 95% CI, 2.206–2.819; *P* < 0.001), 1 extrahepatic site (vs. 0 extrahepatic site, HR, 1.458; 95% CI, 1.262–1.683; *P* < 0.001), 2 extrahepatic sites (vs. 0 extrahepatic site, HR, 2.687; 95% CI, 1.988–3.632; *P* < 0.001) were significantly associated with increased all-cause mortality. Surgery (vs. non-surgery, HR, 0.255; 95% CI, 0.225–0.289; *P* < 0.001); married state (vs. unmarried, HR, 0.661; 95% CI, 0.585–0.747; *P* < 0.001) reduced the risk of death. In the multivariate Cox models, Black race (vs. White race, HR,1.252; 95% CI, 1.049–1.494; *P* = 0.013); American India/Alaskan (vs. White race, HR, 2.325; 95% CI, 1.337–4.044; *P* = 0.003), non-serous type(vs. serous type, HR, 1.651; 95% CI, 1.440–1.892; *P* < 0.001), 1 extrahepatic site (vs. 0 extrahepatic site, HR, 1.168; 95% CI, 1.008–1.352; *P* = 0.038), 2 extrahepatic sites (vs. 0 extrahepatic site, HR, 1.682; 95% CI, 1.238–2.286; *P* = 0.001), 3 extrahepatic sites (vs. 0 extrahepatic site, HR, 2.758; 95% CI, 1.230–6.187; *P* = 0.014) were significantly correlated with a higher risk of all- cause mortality. Surgery (vs. non-surgery, HR, 0.324; 95% CI, 0.278–0.378; *P* < 0.001); married state (vs. unmarried, HR, 0.821; 95% CI, 0.723–0.931; *P* = 0.002) reduced the risk of death. Age at diagnosis, pathological grade and insurance state were not correlated with all-cause mortality.

**Table 4:** Cox regression of OS among ovarian cancer patients with liver metastases.

	**Univariable analysis**		**Multivariable analysis**	
**Variable**	**Hazard ratio (95% CI)**	***P-*value**	**Hazard ratio (95% CI)**	***P*-value**
**Age at diagnosis, y**				
18–40	1 (Reference)		1 (Reference)	
41–60	0.862 (0.638–1.164)	0.333	1.002 (0.740–1.358)	0.988
>60	1.292 (0.967–1.726)	0.083	1.164 (0.868–1.560)	0.311
**Race**				
White	1 (Reference)		1 (Reference)	
Black	1.500 (1.261–1.784)	<0.001*	1.252 (1.049–1.494)	0.013*
Asian/Pacific islander	0.832 (0.647–1.070)	0.152	1.018 (0.788–1.314)	0.892
American India/Alaskan	1.776 (1.027–3.071)	0.04*	2.325 (1.337–4.044)	0.003*
Unknown	0.791 (0.197–3.167)	0.740	0.940 (0.232–3.808)	0.931
**Histology**				
Serous	1 (Reference)		1 (Reference)	
Non-serous	2.493 (2.206–2.819)	<0.001*	1.651 (1.440–1.892)	<0.001*
**Pathological grade**				
I	1 (Reference)		1 (Reference)	
II	1.590 (0.717–3.526)	0.254	1.521 (0.685–3.379)	0.303
III	1.739 (0.822–3.681)	0.148	1.861 (0.879–3.944)	0.105
IV	1.321 (0.619–2.818)	0.471	1.709 (0.800–3.652)	0.167
Unknown	2.831 (1.344–5.965)	0.006*	1.603 (0.759–3.389)	0.216
**Surgery**				
No	1 (Reference)		1 (Reference)	
Yes	0.255 (0.225–0.289)	<0.001*	0.324 (0.278–0.378)	<0.001*
Unknown	0.369 (0.092–1.479)	0.159	0.351 (0.087–1.412)	0.140
**Extrahepatic metastatic sites to lung, bone and brain, No**.				
0	1 (Reference)		1 (Reference)	
1	1.458 (1.262–1.683)	<0.001*	1.168 (1.008–1.352)	0.038*
2	2.687 (1.988–3.632)	<0.001*	1.682 (1.238–2.286)	0.001*
All 3	2.202 (0.985–4.919)	0.054	2.758 (1.230–6.187)	0.014*
Unknown	1.588 (1.257–2.007)	<0.001*	1.223 (0.965–1.550)	0.096
**Marital status**				
Unmarried^a^	1 (Reference)		1 (Reference)	
Married	0.661 (0.585–0.747)	<0.001*	0.821 (0.723–0.931)	0.002*
Unknown	0.748 (0.551–1.016)	0.063	0.770 (0.564–1.051)	0.099
**Insurance status**				
Insured	1 (Reference)		1 (Reference)	
Uninsured	0.984 (0.715–1.355)	0.921	0.982 (0.707–1.363)	0.912
Unknown	1.051 (0.651–1.698)	0.838	1.272 (0.783–2.067)	0.331

a Including divorced, separated, single (never married), and widowed. *denotes a statistically significant P-value.

## Discussion

The dissemination types of ovarian cancer were divided into the transcoelomic metastasis, hematogenous and lymphatic spread metastasis, which individually had distinct molecular metastases mechanisms (10). Some research focused on the mechanisms of distant metastases including liver metastases in ovarian cancer. Kim et al. found the reduction of chemokine receptor the lymphotactin receptor (XRC1) suppressed the colon, spleen and liver metastases of SKOV3-xenograft mouse model (11). A study from Li et al. revealed that high level of insulin-like growth factor-1 (IGF1) was associated with advanced clinical stage and liver metastases of ovarian cancer patients by analyzing the expression of IGF1 in epithelial ovarian cancer clinical specimens. Further basic research manifested the IGF1 promoted the proliferation and migration of ovarian cancer cells and inhibition of IGF1 receptor and the downstream molecules effectively suppressed the malignant phenotype of tumor cells. Therefore, targeting the IGF1 pathway may be promising for the treatment for ovarian cancer patients with liver metastases (12). Joelle et al. found that the cell surface glycoprotein CD44 contributed to the spheroid formation, mesothelial adhesion and mesenteric metastasis in epithelial ovarian carcinoma. However, decrease of CD44 expression promoted the peritoneal metastases like liver and the thoracic cavity (13). Wang et al. found the overexpression of miR-203 attenuated the TGFβ pathway and inhibited the epithelial to mesenchymal transition. And necropsy of the orthotopic ovarian cancer mouse model showed the miR-203 suppressed primary tumor growth and peritoneal metastases including liver and spleen (14). A study from Yang et al. focused on the role of SMAD4 in ovarian cancer development and invasion. Research results showed that knocking out of SMAD4 impaired the vessel endothelial cell tubule formation. Although nude mice experiment indicated the loss of SMAD4 did not influence the tumor growth, it inhibited the barrier integrity in endothelial cell and promoted the ovarian cancer liver metastases (15). Ponnusamy et al. found MUC4 mucin promoted the process of epithelial to mesenchymal transition, and overexpression of MUC4 induced significantly larger tumors and was associated with a higher incidence of metastasis to distant sites including colon, liver and diaphragm (16). Yu et al. found the lysophosphatidic acid (LPA) receptors including LPA1, LPA2, LPA3 were involved the process of tumor proliferation and invasion by regulating VEGF and the cytokines including IL-6 and IL-8. And the overexpression of LPA receptors was associated with distant metastases including liver, kidney and pancreas by necropsy of the SKOV3 xenografts tumors (17).

In our study, the incidence of patients with liver metastases upon initial diagnosis of ovarian cancer was 4.65%. A study from Dauplat et al. showed the incidence of parenchymal liver metastases including the initial diagnosis and later recurrence was 9.4% and the median survival was 5.0 months among the total 255 patients (18). The discordancy may be caused by the difference of study population. Our study showed that old age, Black race, bilateral tumors, non-serous type, high grade, extrahepatic metastases, advanced stage and lymph nodes involvement were risk factors associated with the presence of liver metastases upon initial diagnosis of ovarian cancer. Previous study found that advanced stage, high grade and lymph node involvement were significant risk factors associated with distant metastases (19). The single-institution study of 244 serous ovarian cancer patients manifested that the increasing age, high grade tumor and advanced stage were risk factors associated with the presence of liver metastases (20). Loizzi et al. analyzed the clinical characteristics and survival of 29 ovarian cancer patients with hepatic metastases. Results indicated that 76% patients presented with papillary serous histology and 62% patients presented with poorly differentiated tumors (21). However, No significant difference was seen when compared the distant metastatic patterns for different histologic variants of ovarian cancer in the study from Rose et al. (22).

In our study, the median OS of patients with ovarian cancer was 16 months (IQR, 3–50 months). The subgroup analysis indicated that the patients with non-serous ovarian cancer and more numbers of extrahepatic sites had worse outcome. The multivariate Cox model showed the Black race, non-serous type and extrahepatic metastatic sites were correlated with increased risks of all- cause mortality, which was basically in accordance with previous studies (4, 21, 23). A study from Loizzi et al. revealed the OS among ovarian cancer patients with liver metastases upon initial diagnosis, with liver metastases as first recurrence, with liver metastases as second relapse was 19 months (IOR: 6–23 months), 24 months (IQR: 3–44 months), 10.0 months (IQR: 1–33 months), respectively, and no significant difference was seen among the three subgroups. The patients with liver metastases only had better survival than those with other metastatic sites (median OS: 25 months vs. 8 months, IQR: 9–44 months vs. 1–20 months, *P* = 0.033). And patients with serous ovarian cancer had better survival than those with other type of ovarian cancer (median OS: 23 vs. 8 months, IQR: 1–44 vs. 1–15 months, *P* = 0.005, HR, 2.875, 95% CI, 2.51–3.23) (21).

The treatment for ovarian cancer patients with liver metastases was still uncertain. Our study showed the surgery of primary site reduced the risk of all-cause death (HR, 0.255; 95% CI, 0.225–0.289; *P* < 0.001). A study enrolled in 105 patients with stage IV ovarian cancer from Curtin et al. showed surgery was an important determinant prognosis (24). Gallotta et al. retrospectively analyzed the clinical outcome of laparoscopic secondary cytoreduction for 29 patients with localized recurrent ovarian cancer. The rate of complete debulking was 96.2% and the median DFS was 14.0 months (25). A study of analyzing the safety of laparoscopic secondary cytoreductive surgery in 58 patients with platinum-sensitive recurrent ovarian cancer showed that the median PFS was 28.0 months and the 2 year OS was 90.7% (26). Wang et al. found that the OS of ovarian cancer patients with liver metastases who received R0 liver resection and cytoreductive surgery were 50.1 months, however, the OS of patients who received R0 cytoreductive surgery and non-R0 liver resection was 20.0 months (27). Some selected patients with cytoreductive surgery and liver resection had better outcome. A complete cytoreduction to no residual disease, good performance status, negative resection margins, less numbers of liver lesions and long progression-free interval were significant factors correlated with favorable outcome of ovarian cancer patients with liver metastases. However, it is worth noting that liver resection may cause some relevant complications like bilioma, abnormality of liver function, diaphragmatic injury, chest complications, and bile leakage (28). Transarterial chemoembolization (TACE) had some role in the treatment of ovarian cancer patients with liver metastases. The survival rates after receiving TACE was 58% after 1 year, 19% after 2 years, and 13% after 3 years (29). A study of 109 patients with stage IV ovarian cancer from Giovanni et al. found patients with multiple unresectable liver metastases had worse survival than those with resectable liver involvement (median OS, 14 months vs. NR, *P* = 0.003) (30). In a retrospective study of 37 patients with stage IV epithelial ovarian cancer with liver metastases, Naik et al. found that optimal cytoreduction is an independent prognostic factors associated with more favorable outcome (31). Zhuo et al. analyzed the survival difference of 29 ovarian cancer patients with liver metastases receiving microwave ablation (MWA) or surgical resection (SR). And no significant difference was seen between the two groups (5 year OS rate: SR vs. MWA, 64.3% vs. 51.3%, *P* = 0.198) (32).

Ailbhe et al. compared the clinical characteristics and survival between ovarian cancer patients with liver parenchymal invasion (LPI) from peritoneal metastases and those with hematogenous liver metastases (HLM). Results showed increasing age and suboptimal cytoreduction were factors associated with LPI while increasing age, high grade tumor and advanced stage were risk factors correlated with HLM. Survival analysis showed that ovarian cancer patients with LPI had similar survival to those without LPI (median OS: 80 vs. 123 months, IQR: 50-NR vs. 49–279 months, *P* = 0.6) while ovarian cancer patients with HLM had worse survival than those without HLM (median OS: 63 vs. 145 months, IQR: 43–139 months vs. 50-NR, *P* = 0.006). Therefore, it may be important to elucidate the clear criteria and identify the metastases type among ovarian cancer patients with liver metastases for individual clinical management (20). A study from Charlie et al. compared the frequency of visceral metastases between BRCA1/2 deficient ovarian cancer patients and BRCA1/2 proficient ovarian cancer patients, BRCA1/2 deficient ovarian cancer had increased incidence of visceral metastases (BRCA1/2 deficient vs. BRCA1/2 proficient, 58% vs. 5%, *P* < 0.001) and liver metastases (BRCA1/2 deficient vs. BRCA1/2 proficient, 42% vs. 0%, *P* < 0.001). BRCA1/2 sequencing should be considered among the ovarian cancer patients for better clinical management (33). Gallotta et al. analyzed the survival of 34 recurrent ovarian cancer patients with liver metastases who underwent liver resection within secondary cytoreductive surgery. Results indicated that patients with BRCA mutation had better survival than those with BRCA wild type (3 year post-liver resection progression free survival: 81.0% vs. 15.2%, *P* = 0.001). The assessment of BRCA mutational status may be important for risk stratification among the ovarian cancer patients with liver metastases (34). Sood et al. evaluated the status of p53 mutation in 130 ovarian cancer patients. Results revealed patients with a null mutation had a higher incidence of distant metastases than those with missense mutations or wild type p53 (66% vs. 8% vs. 8%, *P* < 0.001). Twenty five percentage patients with null p53 mutation presented with distant metastases including liver, spleen, brain, and thorax at initial diagnosis of ovarian cancer. It may be important to evaluate the p53 status for clinicians when dealing with the ovarian cancer patients (35).

To our knowledge, this current study was the largest study about ovarian cancer patients with liver metastases. However, several limitations should be acknowledged. First, the information provided by SEER database is insufficient, including the other metastatic sites such as peritoneal metastases of ovarian cancer, the detailed data about size and number of liver metastases and the information of individual treatment. Second, the study population are mainly in the ovarian cancer patients with liver metastases upon initial diagnosis excluding those developed with liver metastases during the recurrence. Third, the SEER database is based on the register in the United States. The study results may cause deviations in the other parts of the whole world.

In conclusion, this present study provided valuable information including incidence, risk factors and prognostic factors for newly diagnosed ovarian cancer patients with liver metastases. These findings assist clinicians make clinical management decisions of prognostic assessment and risk stratification. In the future, basic research and large sample prospective clinical trials are warranted to further evaluate the molecular characteristics and treatment for ovarian cancer patients with liver metastases.

## Data Availability Statement

The raw data supporting the conclusions of this article will be made available by the authors, without undue reservation.

## Ethics Statement

The studies involving human participants were reviewed and approved by The Ethical Committee Review Board of Fudan University Shanghai Cancer Centre (Shanghai, China). Written informed consent for participation was not required for this study in accordance with the national legislation and the institutional requirements.

## Author Contributions

HZ, JL, and XW contributed to the conception and design of this study. HZ, FX, and MN analyzed the data. HZ and XW contributed with a critical revision of the manuscript. All authors contributed to the article and approved the submitted version.

## Conflict of Interest

The authors declare that the research was conducted in the absence of any commercial or financial relationships that could be construed as a potential conflict of interest.
